# AAPM WGWMRSC Report 420: chapter climate check: Mixed methods analysis of survey responses

**DOI:** 10.1002/acm2.14600

**Published:** 2025-02-11

**Authors:** Ashley J. Cetnar, Ghada Aldosary, Meghan C. Koo, Holly Lincoln, Angélica Pérez‐Andújar, Surendra Prajapati, Samantha J. Simiele, Kristi R. G. Hendrickson

**Affiliations:** ^1^ Department of Radiation Oncology The Ohio State University Columbus Ohio USA; ^2^ Department of Radiation Oncology Ministry of National Guard Health Affairs Riyadh Saudi Arabia; ^3^ King Abdullah International Medical Research Center Riyadh Saudi Arabia; ^4^ The Ottawa Hospital Research Institute Ottawa ON Canada; ^5^ Department of Physics Toronto Metropolitan University Toronto ON Canada; ^6^ University of Connecticut Health Center Farmington Connecticut USA; ^7^ Department of Medical Physics Memorial Sloan Kettering Cancer Center West Harrison New York USA; ^8^ Department of Radiation Physics University of Texas MD Anderson Cancer Center Houston Texas USA; ^9^ Department of Radiation Oncology University of Washington Seattle Washington USA

**Keywords:** AAPM, chapters, climate, leadership, professional

## Abstract

**Introduction:**

The American Association of Physicists in Medicine (AAPM) recently shared results and recommendations from its first Equity, Diversity, and Inclusion (EDI) Climate Survey, which was designed to assess the climate at the workplace, the AAPM organization, and the AAPM regional chapter level. This work further explores the status of EDI at the regional chapter level.

**Methods:**

AAPM's EDI Survey was distributed to 5500 members and had a response rate of 25%. In the survey, three open‐ended comment boxes were provided for feedback, including one for regional AAPM members. Sixty‐four percent of respondents indicated they were part of a regional chapter, and 6% provided written responses to the regional chapter question. Responses were analyzed using a mixed methods approach with an exploratory sequential design. Two phases were conducted; the first relied on a Grounded Theory quantitative systemic approach, and the second applied qualitative analysis. Chapter member demographic data were collected to support findings.

**Results:**

Survey respondents provided open comments and feedback on their regional chapter's climate. Data are summarized as five themes: positive experiences, negative experiences, challenges within chapters, diversity and inclusion, and changes observed. Experiences of regional chapters were rated positively by 75% of respondents. Respondents found their chapters were welcoming, and some noted their great chapter leadership. A number of incidents of sexual harassment, bullying, and discrimination incidences were also shared. Other respondents observed exclusion based on their gender, race, highest degree, and medical physics specialty. Chapter leadership data aligned with these claims, with most leaders to‐date being white males, doctoral degree holders, and/or specializing in radiation therapy.

**Conclusion:**

AAPM chapters provide rewarding professional opportunities. This study has highlighted positive and negative experiences reported by its members. The major themes identified can guide chapter leaders to continue to cultivate welcoming communities for regional AAPM members.

## 1 INTRODUCTION

The American Association of Physicists in Medicine (AAPM) is a scientific and professional organization with currently 9797 members in 94 countries. 86.6% of AAPM's members are working in the US, 4.0% in Canada, and 9.4% in other countries.^1^ AAPM's mission is to advance medicine through excellence in the science, education, and professional practice of medical physics, which is an interdisciplinary field utilizing the principles of physics, biology, and medicine.

The climate of an organization was characterized in the survey by perceptions of inclusion (welcome), respect, opportunities, and recognition within the workplace, as well as reports of experiences with negative behaviors such as exclusion, intimidation, hostility, and microaggressions. Surveying members’ attitudes and experiences about the organization's climate is valuable for assessing the health of the organization from the perspective of membership. As a result of the AAPM's adoption of diversity and inclusion as a strategic goal,^2^ the first Equity, Diversity, and Inclusion (EDI) Climate Survey was sent to medical physicists at a national level in 2021, providing valuable baseline data for full members of the organization.^3^ The three goals of the climate survey were to assess the climate for full members at the level of (1) the individual's workplace, (2) AAPM as an organization, and (3) regional chapter. Here, we focus on the status of EDI at the regional chapter level.

**FIGURE 1:**
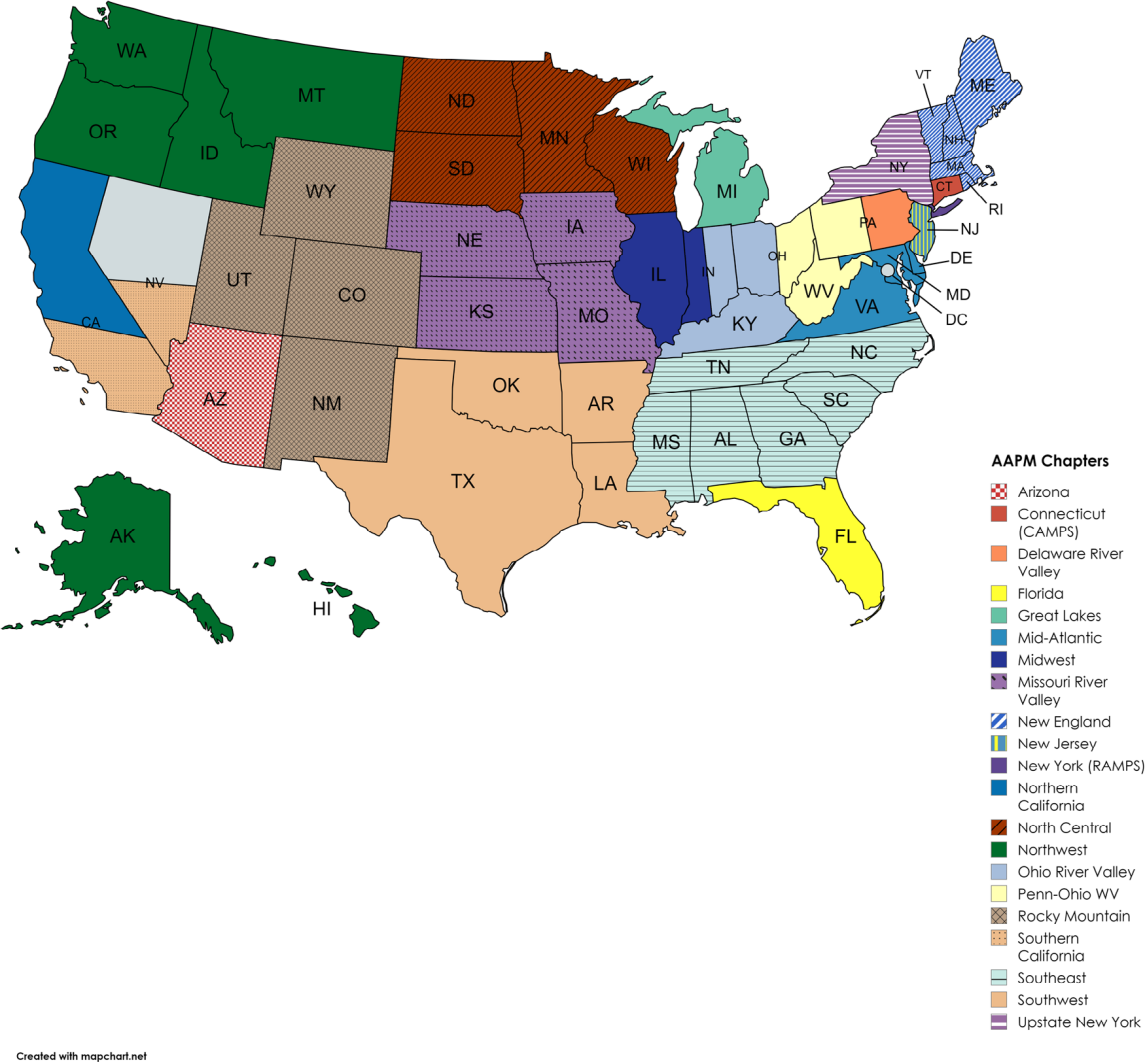
Representation of AAPM chapter map^4^. AAPM, American Association of Physicists in Medicine.

Chapters function under the sponsorship of the AAPM and have memberships ranging from 130–770 individuals composed of medical physicists, students, trainees, and potentially other professional affiliates. Regional chapters offer continuing education opportunities, scientific meetings, networking opportunities with colleagues, meetings with vendors, and a pathway to leadership within the chapter. The 21 regional chapters of the AAPM have independently determined governance.^4^ Figure 1 includes a representation of the chapters created by the authors based on the AAPM chapter map reference, but boundaries within states may not be accurately depicted based on limitations of the software. Chapter constitutions, bylaws, and rules are similar among chapters but are not required to be identical to a template offered by the national AAPM. Chapters cannot have constitutions, bylaws, or rules that conflict with AAPM rules. All chapter bylaws are available on the AAPM website,^4^ and most bylaws are also available on individual chapter websites for reference. Each chapter is unique and has its own professional climate.

Regional chapters are incredible opportunities for professional growth, networking, and leadership development. Most members experience positive relationships with their regional chapter organizations. However, there are certain behaviors or systems that can be a barrier to members feeling welcome and included in their chapters. While these circumstances may not be present in all chapters, the goal of this report is to expose specific challenges experienced by members to promoting awareness of issues so that climates in Regional Organizations (ROs) can continuously improve. In this study, we present the 2021 AAPM EDI Climate Survey open‐ended response data from members of the organization regarding the climate in AAPM regional chapters.

## 2 METHODS

Survey methods have been described in our published executive summary.^3^ In brief, the 2021 AAPM EDI Climate Survey was designed by a working group of the AAPM Diversity and Inclusion Subcommittee. AAPM partnered with the Statistical Research Center of the American Institute of Physics (AIP) to implement the survey, targeting medical physicists who are full members of the AAPM. The survey included demographic inquiries and questions intended to assess the working environmental climate in terms of a sense of belonging and inclusion, experiences of discrimination and harassment, and obstacles to participation within the AAPM. The survey invitation was sent to 5500 members, and responses were collected from 1385 members (response rate of 25%) between January and February 2021. Three open‐ended comment boxes were provided for additional thoughts, feedback, or recommendations, including one for regional AAPM chapters. Of the 1385 respondents, 64% (883/1385) indicated they were part of a regional chapter.^3^ However, many of the participants in the survey did not respond to the open‐ended question regarding regional chapters, where 6% (57/883) provided a written response to this question.

In this study, a mixed methods approach was used with an exploratory sequential design. The first phase of the approach applied qualitative methods, and the second phase applied quantitative methods built on the results of the first phase.^5^ Qualitative research is focused on answering questions of “why” and “how,” while quantitative methods help to answer the “what” questions. Survey written responses were evaluated using a Grounded Theory systematic approach as defined by Charmaz.^6^ This approach provides “systematic, yet flexible guidelines for collecting and analyzing qualitative data to construct theories ‘grounded’ in the data themselves.^6^” Qualitative data analysis was performed using Dedoose Software (version 9.0.86, Los Angeles, CA). Line‐by‐line coding was used to develop axial codes and then theoretical codes. Interrater reliability was maximized among multiple coders (*n* = 3) through frequent meetings after independent coding to discuss and reach consensus on code definitions and coding of excerpts from comments by the larger committee. While all survey data are anonymous, to respect respondents’ privacy, statements and experiences are not reported in this publication with direct quotations.

Based on the results of the qualitative analysis, additional research questions were asked to establish the context of major themes related to chapter leadership and trends. A database of demographic data from chapter leaders was created to review historical chapter leadership data. Officers of the chapters are elected following the chapter bylaws. Each chapter also elects or nominates a representative to the national AAPM Board of Directors, adding 21 Board members to 12 elected Board Members‐at‐Large and five elected officers.^7^ The chapter representatives of the AAPM Board also comprise the RO.

This included data from the past 10 years of chapter presidents (2013–2023) and past five board representatives (3‐year terms with different rolling starts per chapter). Because of the variation in additional officers between the regional chapters, other roles (e.g., secretary, treasurer) were excluded for the scope of this study. Demographic information included years in leadership, gender, specialty, highest degree, and institution for chapter leaders. To have a comprehensive and complete database for all areas of interest, sources for the data included past chapter history posters in the AAPM Heritage and History webpage,^8^ individual chapter websites, or institutional websites. Additionally, historical data coinciding with the leadership term was obtained from the AAPM membership database or by request from AAPM staff. The accuracy of this demographic data were verified by current chapter leaders via email with responses from 16 of 21 chapters and additional verification of data accuracy. The database contains 335 total chapter leaders, including 231 chapter presidents, representing a complete data set for the leaders over a decade. With the exception of the Arizona Chapter, which only had four board representatives in its history, 104 chapter board representatives were also included, representing a complete data set for the leaders over a decade. Because term limits vary between chapters, for the purpose of this study, each 1‐year presidential term and 3‐year board term was assigned a unique identifying number. Notably, the same person could hold more than one position over time.

The AAPM Member database was used as a reference for self‐reported demographic data, including specialty, gender, and institution. For each known chapter leader, the primary specialty (i.e., Radiation Oncology, Diagnostic Imaging, Nuclear Medicine, and Radiation Safety) was used to associate each leader with their specialty (therapy, imaging, Nuclear Medicine, and Radiation Safety). “Multiple” was designated if there was not a majority designated for one specialization category in the member profile. If more than one gender option was selected, the primary gender in the database was used in the reported data. Since healthcare institutions and systems may change over time with mergers, rebranding, and so forth, to maintain consistency, the name of the institution was listed as it was referenced in the year of the leadership. Furthermore, because leaders may also relocate between institutions during a leadership term, their affiliated institution from the first year of their leadership term was recorded.

## 3 RESULTS

Survey respondents provided open responses to the questions related to the climate of their chapter. The perceptions and experiences of the members are summarized in the following major themes: positive aspects, negative experiences, challenges of chapters, diversity and inclusion, and changes in climate.

### 3.1 Positive aspects of regional chapters

Although 75% (662/883) of respondents reported that their regional AAPM chapter was welcoming (agreed or strongly agreed),^3^ only 39% (22/57) of the responses to the open‐ended questions on regional AAPM chapter climate had positive attributes. Most of the open‐ended positive responses described atmospheres with sentiments such as good or great chapters, climates, and environments. Others shared in the comments that their chapters were welcoming, did not encounter issues or problems within their chapter, or loved and sincerely appreciated their regional chapter.

Some responses specifically addressed chapter leadership. One respondent said they have great chapter leadership, and another mentioned that they have diverse leadership now compared to how it was in the past. Several responses described the regional AAPM chapter as being inclusive and disclosed that everyone is treated equally and fairly without gender or race bias. One respondent specifically appreciated the inclusiveness and many opportunities for new physicists to get involved in their regional AAPM chapter. Two respondents stated that they do not feel discriminated against within their chapter. Two additional respondents provided optimistic sentiments that their chapters’ climates have changed over time and are getting better slowly.

Most positive responses did not provide specific details on their experiences, even though a few particularly praised the chapter leadership, expressed feelings of inclusiveness, and shared statements that they did not experience discrimination within their chapter. When considering the results, it is important to consider survey response bias. Individuals who feel strongly, either positively or negatively, toward the topic tend to be those who respond to the survey invitation.^9^, ^10^ Within this survey, we note that positive responses in the open‐ended questions were either absent or brief, lacking specific details.

### 3.2 Negative experiences

Not all respondents reported positive experiences. Some individuals shared personal anecdotes that helped identify areas in need of improvement within the chapters. Concerns were expressed related to (1) harassment and unprofessional behavior, more specifically, (2) sexual harassment and bullying, and (3) discrimination based on one's level of highest earned degree.

The smaller and more relaxed environment of the chapter meetings compared with the larger and more formal annual meetings may result in more lenient attitudes of attendees and give the illusion that unprofessional behavior is accepted at these events. One respondent suggested that chapter meetings can feel more informal than national meetings, and because of this perception, attendees may be more comfortable making inappropriate statements. An example provided was a chapter meeting attendee making comments based on gender stereotypes.

Multiple individuals reported that the sexual harassment that they or others have experienced has negatively impacted attendance at chapter meetings. A respondent shared a concern that there is a “creeper” in their chapter who has a history of inappropriate behavior with other members. Another physicist shared that they no longer attend chapter meetings because of instances of bullying and witnessing sexual harassment of others. Paradis et al. recently presented the results of a semi‐structured interview of faculty, staff, and residents at academic institutions.^11^ The work focused on the experiences and perceptions of both man and woman physicists on gender‐based discrimination and harassment within the field of medical physics. Some of the experiences shared by the participants in the work of Paradis et al. have a striking resemblance to the free response comments in this work, with mentions of a “creepy guy” who negatively impacts the professional environment.^11^

Discrimination based on the level of highest degree earned was identified as a theme in this work. In addition to some physicists perceiving chapter membership as preferentially supporting physicists with doctoral degrees for leadership positions, some individuals reported a sense of feeling unwelcome or less valued as a result of holding a master's degree rather than a doctoral degree. One individual shared that there is intentionality in promoting those with doctoral degrees. Another respondent recounted that an officer boasted to them about their status of obtaining a doctorate.

### 3.3 Challenges of chapters

#### 3.3.1 Unwelcoming and exclusive

Based on participant responses, not all physicists believe they are considered equal within their chapters. Comments included that their leaders are primarily represented by therapy physicists, and diagnostic physicists do not have representation in leadership or a voice in content development for meetings. Some respondents also suggested that there was a strategy to promote physicists with doctoral degrees to leadership positions at the chapter level. Respondents shared that one or a few institutions can monopolize individual chapters. To investigate these claims, we report the following data from chapter leaders.

**FIGURE 2:**
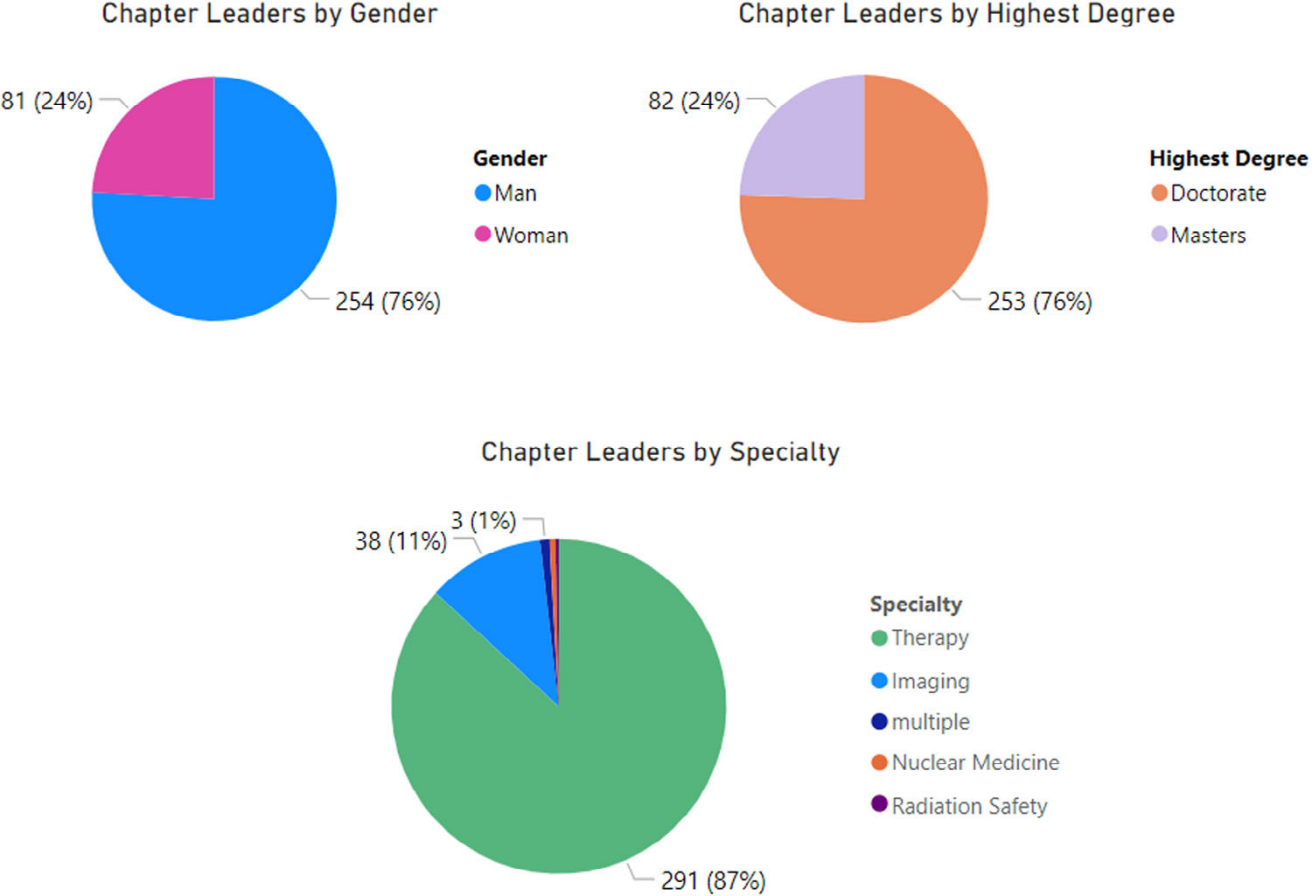
Summary of leader statistics for chapters by gender, specialty, and highest degree.

**FIGURE 3:**
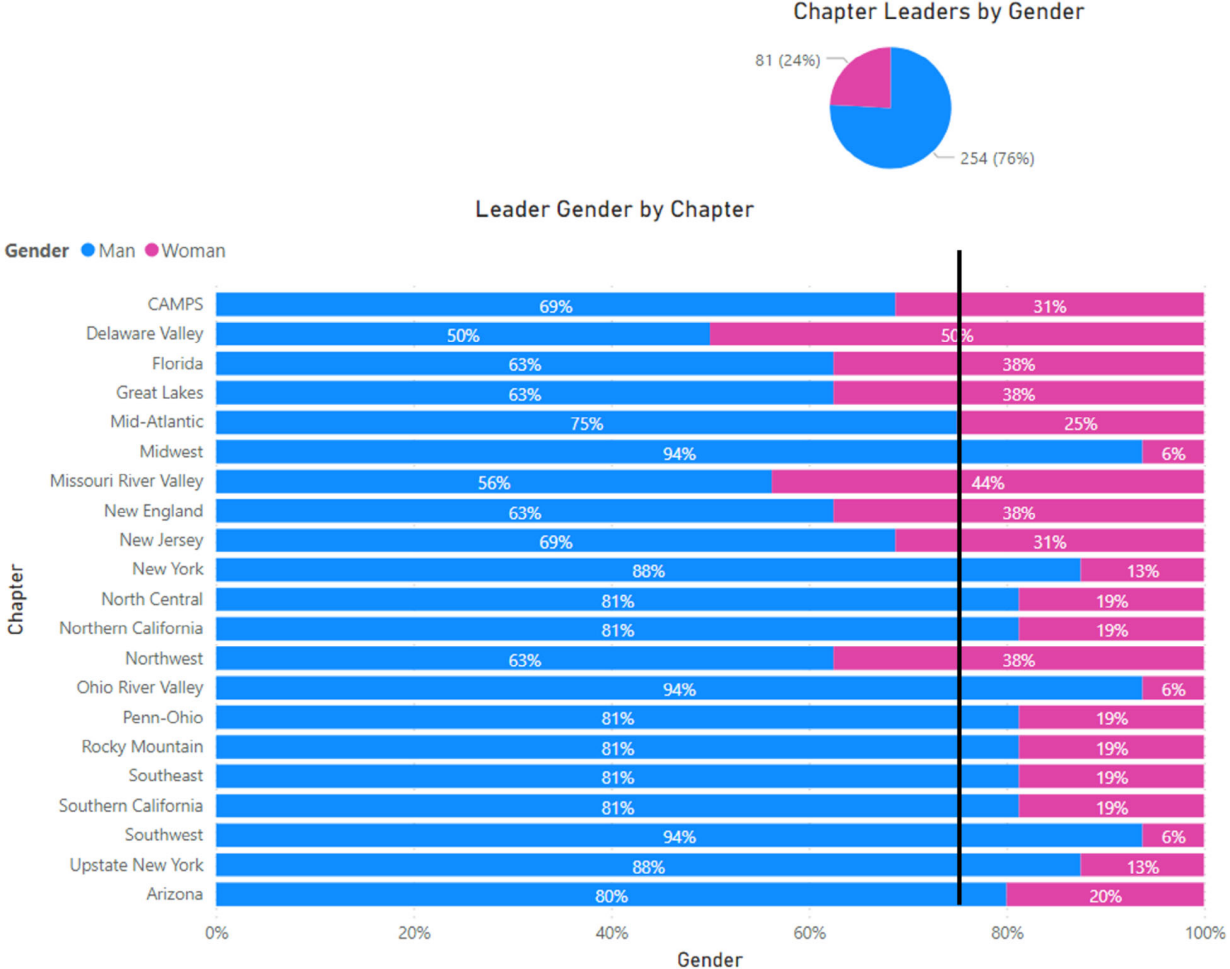
Self‐reported gender of leaders by AAPM chapter. The black benchmark line represents the 75% of AAPM full members who identified as being a man. AAPM, American Association of Physicists in Medicine.

**FIGURE 4:**
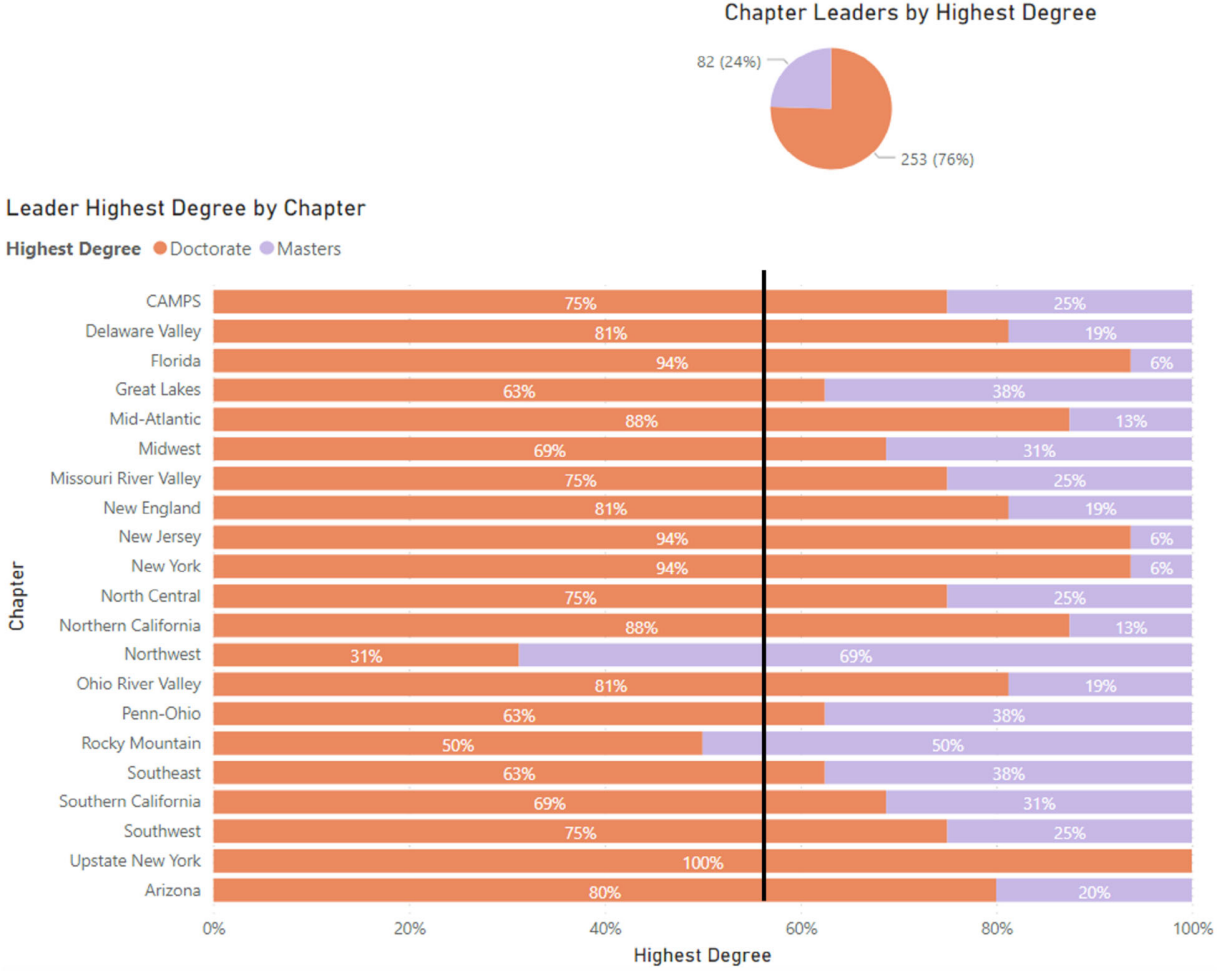
Highest degree of leaders by AAPM chapter. The black benchmark line represents the 57% of the reporting AAPM full members who disclosed a doctoral degree as their highest degree.AAPM, American Association of Physicists in Medicine.

**FIGURE 5:**
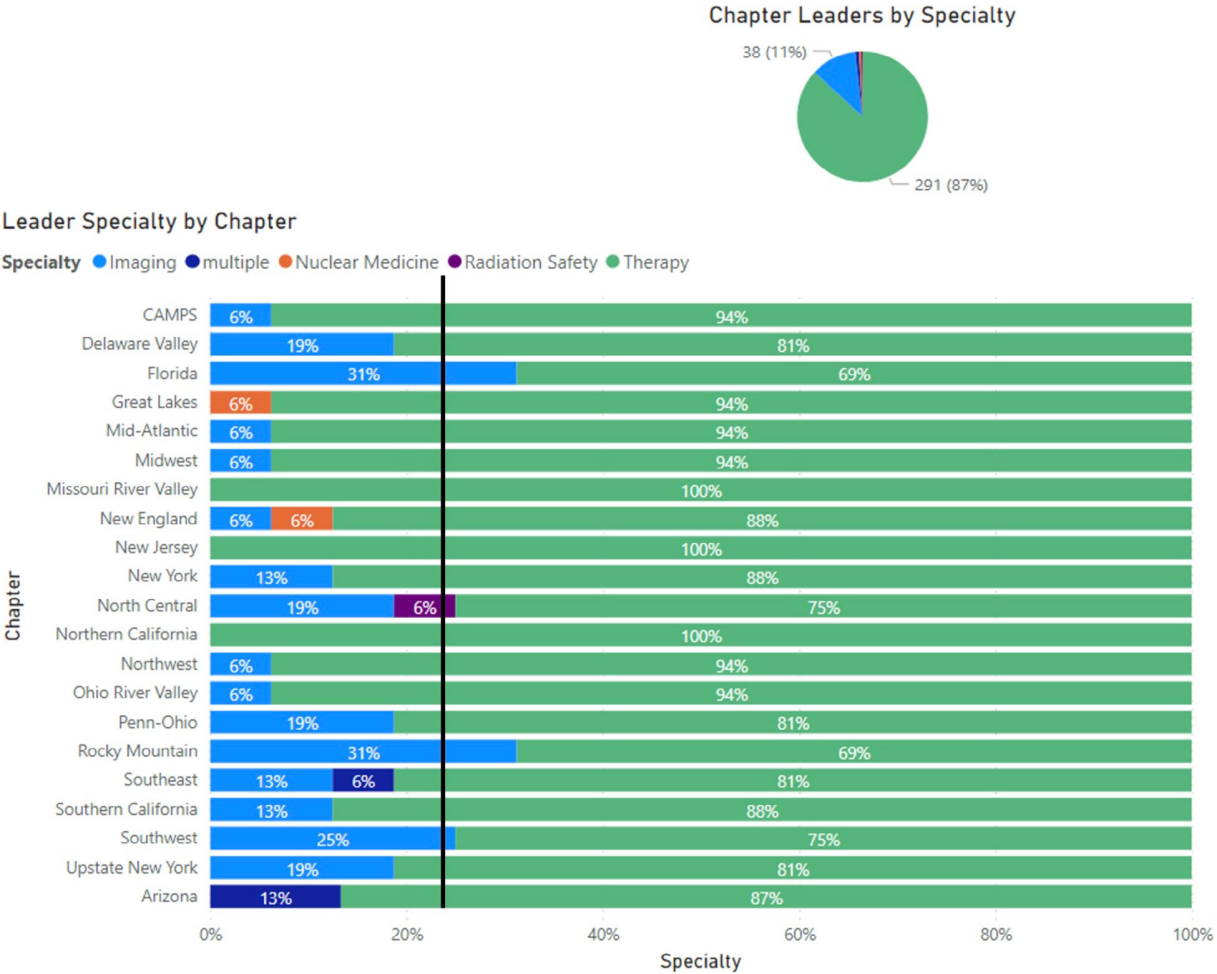
Specialty of leaders by AAPM chapter. The black benchmark line represents the 77% of reporting AAPM full members who specified therapy as their primary specialty. AAPM, American Association of Physicists in Medicine.

Eighty‐seven percent of leaders identify therapy as their primary specialty, 76% of leaders identify as a man, and 76% of leaders hold a doctoral degree as their highest degree (Figure 2). Figures 3, 4, 5 show the breakdown of demographic representation by chapter, contrasted with the percentage of reporting Full AAPM national members for each demographic category. Variation among representations between chapters is observed. Representation of women in leadership positions ranges from 6% to 50%, Master's degree holders from 6% to 69%, and imaging as a primary specialty from 0% to 31%. Additional chapter‐related leadership data can be found in the Supplementary Materials, which compare chapter leadership to membership statistics as benchmarks.

**FIGURE 6:**
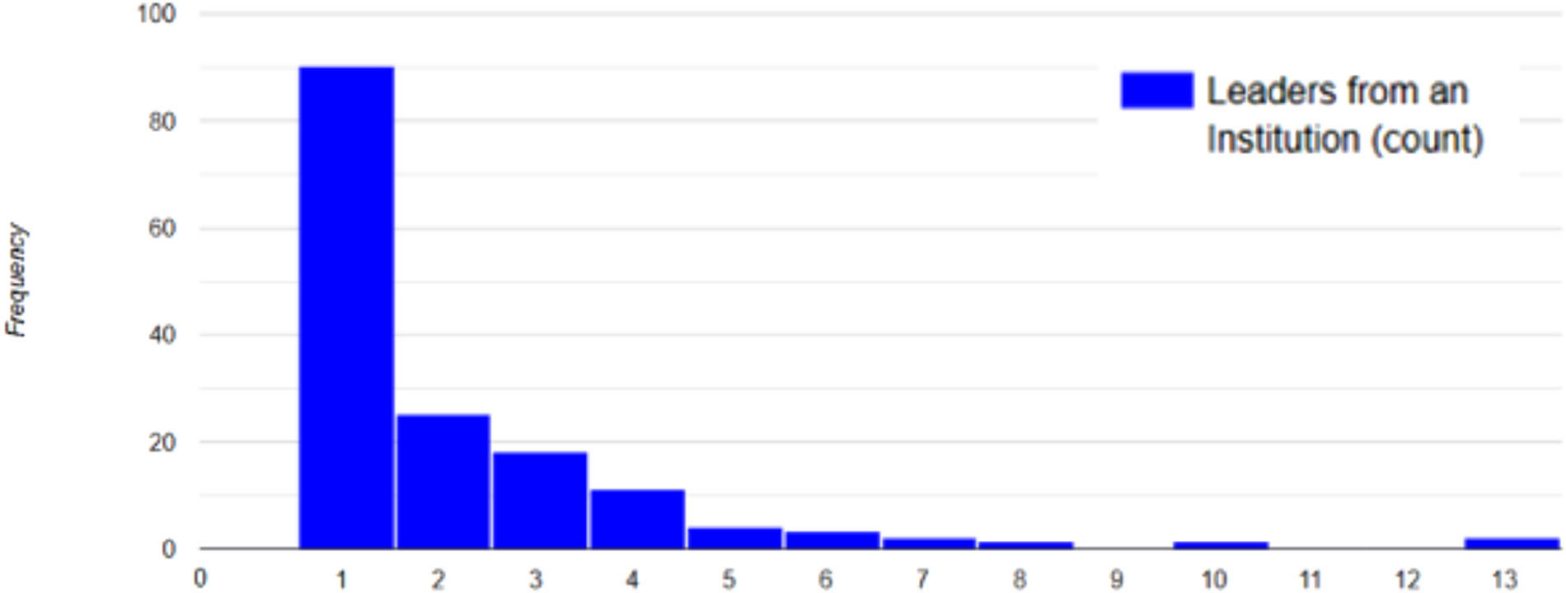
Histogram of number of chapter leaders per institution from database ranging from 1 leader from a single institution to 13 leaders from an institution.

“Elite” is considered a descriptor of a highly selective institution or an entity that limits its access.,^12^, ^13^ Only a few institutions can be considered elite among all institutions of the same type, that is, academic centers. It is also associated with the amount of prestige these centers have in comparison to others. People also associate the use of the word elite with best.^14^ In this context, it is not necessarily associated with the patient access to the institution but with how the members of these institutions may lead the medical physics field and the majority representation within regional chapters. The descriptor of institutions as elite can also show a perception of inequality among institutions. The histogram in Figure 6 reveals that the maximum number of leaders from the same institution is 13 (*n* = 2) out of the 16 possible leaders from a chapter within the time frame sampled in our database. While elite may have different definitions depending on the context, there could be a correlation between institutional representation in leadership at the chapter level and perceptions of elite representation within the regions.

#### 3.3.2 Boys club

From the survey data, several respondents (*n* = 4) expressed that chapters felt like an “old boys club.” This concept is not unique to medical physics and has been researched in fields such as business and economics, environmental sciences, and public health.^15^ The term “old boys’ club” originated from the informal social networks of British elite who attended certain public schools together. Currently, the term refers to the preservation of social elites more broadly.^16^ Male employees have an alleged advantage over their female counterparts in the accessibility of interacting and networking with powerful men.^16^ This results in a self‐perpetuating cycle, or a male‐to‐male advantage, where men in leadership are more likely to promote men who continue promoting other men.^16^ Within academia, prior studies have shown that women account for at least half of the junior faculty. However, female faculty members are less likely to be tenured or promoted compared to their male colleagues, resulting in the decreasing proportion of women to men at the same faculty rank with increasing rank.^17^

This is consistent with the sentiment of several survey respondents who expressed that AAPM chapters are unwelcoming for women, where older, white, male physicists dominate the discussions and conversations. In fact, there were recommendations from experienced chapter members to new members to not be involved in chapters due to the “old boys club” within the survey responses. Several members expressed concern that within chapters where the leaders all know each other, there has not been a history of women being elected to office within the chapter. Some respondents were concerned by a lack of visible efforts to promote diversity and inclusion at the chapter level.

#### 3.3.3 Transparency

All bylaws are available for reference on the national AAPM website for chapters.^4^ Some but not all chapters also make their constitutions and bylaws available on chapter websites. It is unclear if chapter members know where to access bylaws, are familiar with their contents, and whether officers of chapters follow their bylaws based on responses in this survey. Rules for officer nominations and elections differ by chapter and are detailed in the chapter bylaws. Several chapter members expressed concerns about a lack of consistency and transparency in the nomination process for new leaders. Officers are also listed for each chapter on the national AAPM website. However, this list can be inconsistent with individual chapter websites since information may not be updated regularly, making it unclear which reference is accurate.

### 3.4 Diversity and inclusion in chapters

The value of diversity and inclusion in various healthcare professions has been examined through many perspectives.^18^, ^19^ In the workplace, it is recognized that EDI can improve productivity, develop creative problem‐solving, and promote innovation.^20^ In this study sample, participants highlighted the importance of EDI in various workplace opportunities. One participant commented that diversity is important for productive discussion. In theory, diversity is thought to spur productive discussions because different individuals can offer different perspectives. However, according to research, this is only the case if team members are open to differing views and are accepting of different people (i.e., are inclusive) and provide equitable opportunities for those people to contribute to the team.^21^

In the 2021 AAPM EDI Climate Survey,^3^ 75% (657/876) of AAPM Full Members identified as being white and 61% (845/1385) as men. If, as an example, we take race/ethnicity and gender as the predominant descriptors of demographic homogeneity, we note that to this day, the majority of AAPM chapter leaders have self‐identified as men (76%: 196/258), as shown in Figure 3. This lack of demographic diversity has not been unnoticed by chapter members. For example, one study participant noted a lack of leadership awareness when there was a lack of diversity in invited speakers in a meeting program. Another participant shared that they believed the lack of diversity within chapters may stem from implicit bias rather than direct intent. The respondent shared that the leaders were not intentionally discriminating but were not aware of or actively pursuing ways of being more inclusive within the chapter.

While experiences may vary across different chapters, recent data shows that gender representation is growing within the AAPM community.^22^ One participant reflected on this change, expressing difficulty in contributing new perspectives amid conversations that were dominated by a small group of members. However, it was believed that leadership is changing with hopes for cultural change within the chapter. With the changing demographics of North American (U.S.^23^, ^24^ and Canadian^25^, ^26^ ^)^ populations in the next decade, we should also aim to see more facets of diversity present within Full AAPM members (e.g., socioeconomic status, religion/faith, disability, sexual identity, etc.).

AAPM members and leaders have advocated for EDI and continue to invest resources towards providing awareness and education to cultivate it. While AAPM Leadership continues to champion these initiatives, in AAPM chapters, EDI work still encounters several challenges. While some chapters are strategically integrating EDI into their climates, one such challenge is lack of engagement among chapter leaders and members. A respondent shared that there was a meeting focused on diversity and inclusion, but it was not as well attended as other chapter meetings. While efforts to provide education in diversity and inclusion have taken place and have been beneficial to attendees, not all members will choose to attend a meeting where this topic is the primary focus. Another challenge encountered is individuals devaluing diversity and inclusion within chapters based on negative comments shared by chapter members during meetings. These opinions and observations are not unique to the medical physics culture and have been reported by other health professions.^27^

### 3.5 Changes in climate

Several respondents expressed their hope for more inclusive chapter environments in the future. Individuals acknowledged that local chapters have not always been welcoming to everyone, but improvements have been witnessed in recent years.

AAPM will need to be innovative in approaches to address EDI at the chapter level, as there are few organizations within the field of Radiation Oncology and radiology that have regional chapters from which we can learn. The American Society for Therapeutic Radiation Oncology (ASTRO), the Radiological Society of North America (RSNA), and the Canadian Organization of Medical Physicists (COMP) all exist at the national level and do not have independent, smaller subunits for their members. The American College of Radiology (ACR) does have local chapters, but this research team was unable to find information on the status of EDI within the chapters nor identify explicit efforts to address EDI at the chapter level.

There is evidence of change in culture at the national level for many organizations within the field of medical physics. Two recent publications detailed the status of EDI within AAPM^3^ and COMP,^28^ arising from organization‐level effort, demonstrating that leadership within these organizations is attentive to the topic and investing resources to collect baseline data. It will take years for current and future efforts to increase EDI and shift the culture, and longer to administer surveys and collect data to quantify improvements. Until then, initiatives to improve EDI at the national level must continue, while initiatives at the chapter level must be developed and implemented.

## 4 DISCUSSION

Regional chapters can be vibrant organizations for professional development and engagement. While most chapters are currently welcoming and inclusive, we recognize there are always ways for growth and improvement within organizations. In the process of validating the data for this survey, the authors have been able to correspond with current regional leaders about chapter climate. As a result, we are currently involved in collaborative and respectful discussions in supporting chapters in the implementation of strategies for improving the climate for all members. Being mindful of the climate of the chapters is beneficial for increased participation and engagement of members. Considering ways to improve chapter climate can include (1) adoption or development of policies at the chapter level, (2) guidance from the RO committee, (3) commitment from chapter leadership, and (4) engagement from chapter membership.

1. In 2015, the AAPM issued a Diversity Statement, which was later updated in 2020.^29^ The purpose of this statement is to promote an inclusive environment within the organization. Individual chapters could work collaboratively with the AAPM to adopt the policy and include the verbiage in chapter bylaws.
2. The RO is a committee under the Administrative Council that is charged with fostering communication among and between chapters and the Board of Directors of the AAPM. Additionally, the RO provides a forum for chapter concerns and questions and to be a resource of information for chapters. Membership of the RO consists of the Chapter Representatives from each of the 21 Regional Chapters and are elected as per individual Chapter By‐laws. As with all AAPM Board of Directors, Chapter Representatives are responsible for the affairs of the AAPM, including having an impact on current matters and contributing to the strategic plan of the organization.
3. Individuals in current and future chapter leadership roles, such as Chapter Representative, have the unique opportunity to address the climate within their own chapter and to have a meaningful impact. It is important for chapter leadership to realize that although the comments reported in the 2021 AAPM EDI Climate Survey were anonymous and cannot be traced back to specific chapters, incidents could be present or possible within all chapters and thus, require proactiveness. It is hoped that leaders will be able to reflect on the examples provided in this report to strategically plan how challenges can be addressed systemically or in response to instances that could occur within the organization. Four opportunities for fostering a more inclusive environment include (a) actively engaging with new members, (b) encouraging diversity in leadership roles, (c) transparency in operations, and (d) developing a variety of leadership skills to prepare for the non‐technical aspects of leading a chapter.
4. Multiple approaches may be used to engage and welcome new members. One respondent suggested new members could be assigned a mentor for the meeting. Some potential goals of this mentorship relationship could be to learn more about the history and current initiatives of the chapter, provide introductions to other members, and be available to answer questions for the new member. Another option for providing a welcoming environment would be incorporating a new member session into the chapter meeting, similar to the New Member symposium offered at the AAPM annual meeting to individuals who have joined the association within the last 5 years. This session would be an opportunity for new members to meet their chapter leadership and learn about the resources available to members. A third possibility is a mentorship program for students and trainees that could mirror mentorship programs offered at the national level.

Multiple respondents reported minimal turnover in the makeup of their chapter leadership, resulting in stagnant operations and increased feelings of a “boys club.” This commentary begs the question of how to encourage greater diversity in leadership. We encourage all members to engage proactively as volunteers within their chapter and express interest to leaders within their chapters in how to be involved. There are some leadership roles at the national level (e.g., AAPM President) that historically alternate between specialties (e.g., therapy and imaging) each year, as an intentional way of promoting representation of sub‐specializations at the highest level of the organization. A similar approach could be taken at the chapter level, with the officer positions being recruited from different degree levels (MS and PhD), gender, and hospital affiliation (academic or community). Additionally, the number of terms could be limited to one for each position type to better distribute leadership opportunities among chapter membership. The development of additional bylaws should be thoughtful, given each chapter has a smaller candidate pool at the regional level than the national level and there may be existing cultural precedent for filling these roles, where it may be difficult to always satisfy strict rotation policies.

The AAPM implemented the Medical Physics Leadership Academy (MPLA) to provide physicists with tools needed to develop leadership skillsets that are often overlooked or not included in the curricula of training programs.^30^ The MPLA recently created a Leadership Handbook with the intent to provide helpful information for individuals chairing groups within the national organization.^31^ The RO could adopt this resource or create an equivalent handbook for chapter leadership, offering suggestions for improving the climate at the local level.

Opportunities for invoking change exist also at the level of the individual. One respondent offered that chapters’ environments are more volatile and variable depending on the size and climate of each individual chapter. The relatively small size of the chapters could result in change at a rate faster than what is possible at the national level. Fewer total individuals, passionate about changing the culture, would be needed to form a critical mass and make improvements at the regional level. Each individual member is responsible for familiarizing themselves with the bylaws of their chapter, knowing their rights as members, and advocating for change.

While the goal of the study is to provide examples from members in which we can reflect and learn, there are limitations to the study. The sample size is small for those who completed open response questions (*n* = 57). The themes presented in this work are not meant to be generalizations of the climate in chapters as a whole but provide awareness of aspects that could be harmful to members. The responses of the individuals in this study are valued but are limited to those who completed the survey, so full context is not available for every situation presented. Within this study, we did not include data for additional leadership positions beyond president and board representative, such as treasurer and secretary. All leaders within the chapters can make a significant impact on the climate of the organization, but these officers were excluded from this review because of differences between chapters in the number and types of additional officers included in executive committees. Demographic information reported in this study is confined to the leaders, but future work can include the study of the demographic information for chapter membership and analyze changes in leadership from medical physicists from other demographic aspects such as underrepresented minorities. For climate surveys in the future, we would like to include additional language for consent from respondents to be able to share direct quotations from the participants so that experiences would not need to be generalized for future reporting.

## 5 CONCLUSIONS

AAPM chapters provide an incredible opportunity for local members to engage professionally. While some respondents have shared positive experiences as a member of their local chapter, there are also many potential challenges to creating a welcoming climate at the local level. We hope the data from this report will be useful to chapter leaders in continuing to cultivate welcoming communities for medical physicists.

## AUTHOR CONTRIBUTIONS

**Ashley J. Cetnar**: Conceptualization; methodology; investigation; data curation; writing—original draft; visualization. **Ghada Aldosary**; **Meghan C. Koo; Angélica Pérez‐Andújar**; **Surendra Prajapati**; **Samantha J. Simiele**; and **Kristi R. G. Hendrickson**: Investigation; data curation; writing—original draft; writing—review and editing. **Holly Lincoln**: Investigation; writing—review and editing

## CONFLICT OF INTEREST STATEMENT

The authors declare no conflicts of interest.

## Supporting information



Supporting Information
