# Correction to: “AAPM WGWMRSC Report 420: chapter climate check: Mixed methods analysis of survey responses”

**DOI:** 10.1002/acm2.70131

**Published:** 2025-06-11

Cetnar AJ, Aldosary G, Koo MC, et al. AAPM WGWMRSC Report 420: chapter climate check: Mixed methods analysis of survey responses. *J Appl Clin Med Phys*. 2025; 26:e14600.

The original article omitted the AAPM Report and Working Group information. The corrected title is, “AAPM WGWMRSC Report 420: chapter climate check: Mixed methods analysis of survey responses”. Further, the article type has been updated to reflect that this is an AAPM Report & Documents.

