# Supplementary material for: AAPM WGWMRSC Report 420: chapter climate check: Mixed methods analysis of survey responses
Source: J Appl Clin Med Phys. 2025 Feb 11;26(3):e14600. doi: 10.1002/acm2.14600 (PMC11905241; doi:10.1002/acm2.14600)
Supplement: Supplementary file 1 — Supporting Information [file ACM2-26-e14600-s001.docx]

# Supplemental Data

## Data by Presidents


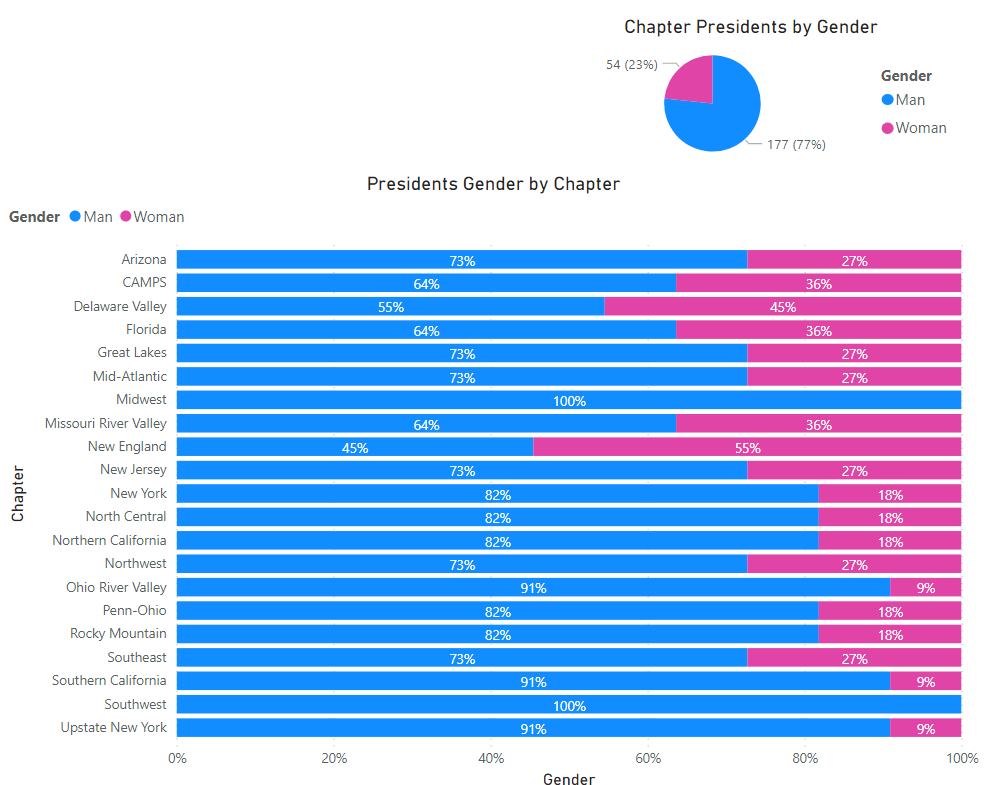


Figure 7: Gender of chapter presidents by AAPM chapter from 2013-2023


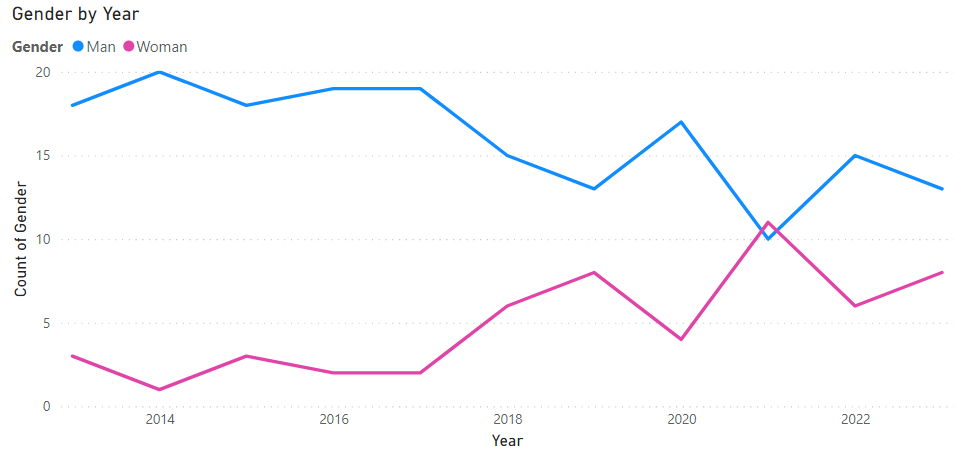


Figure 8: Chapter trends in gender over time for chapter presidents


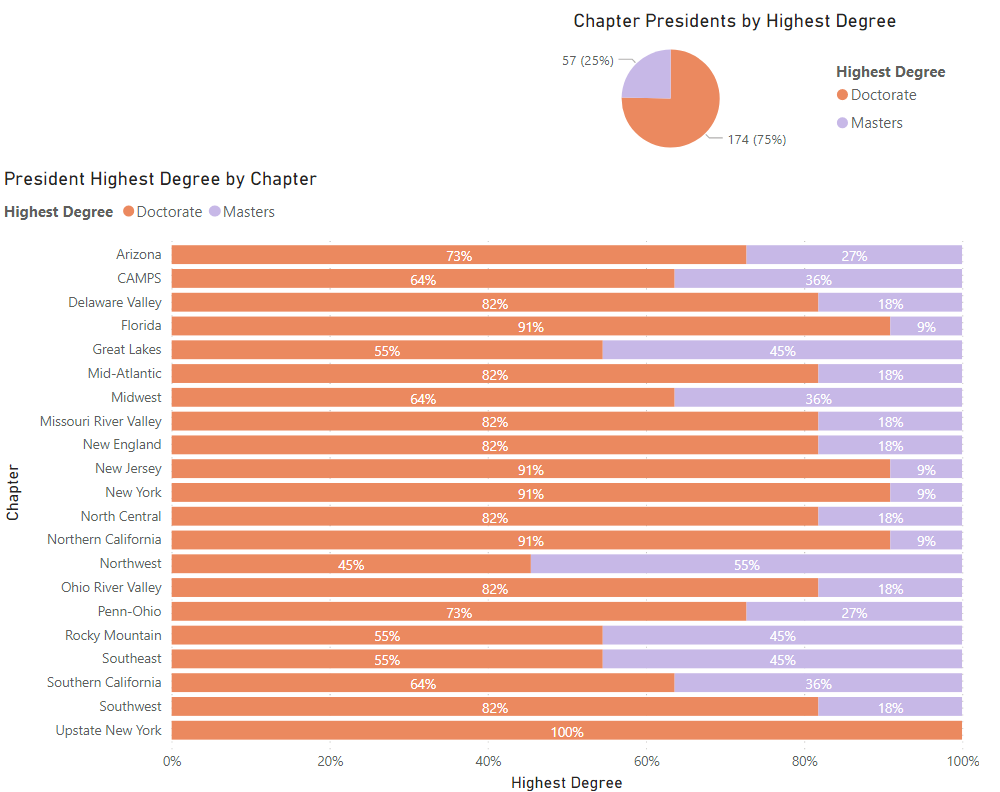


Figure 9: Highest degree of chapter presidents by AAPM chapter from 2013-2023


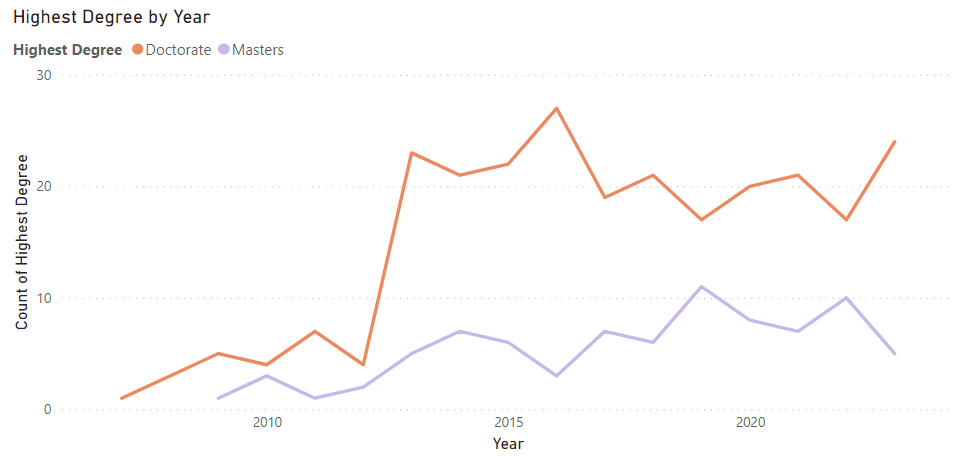


Figure 10: Chapter trends in highest degree over time for chapter presidents


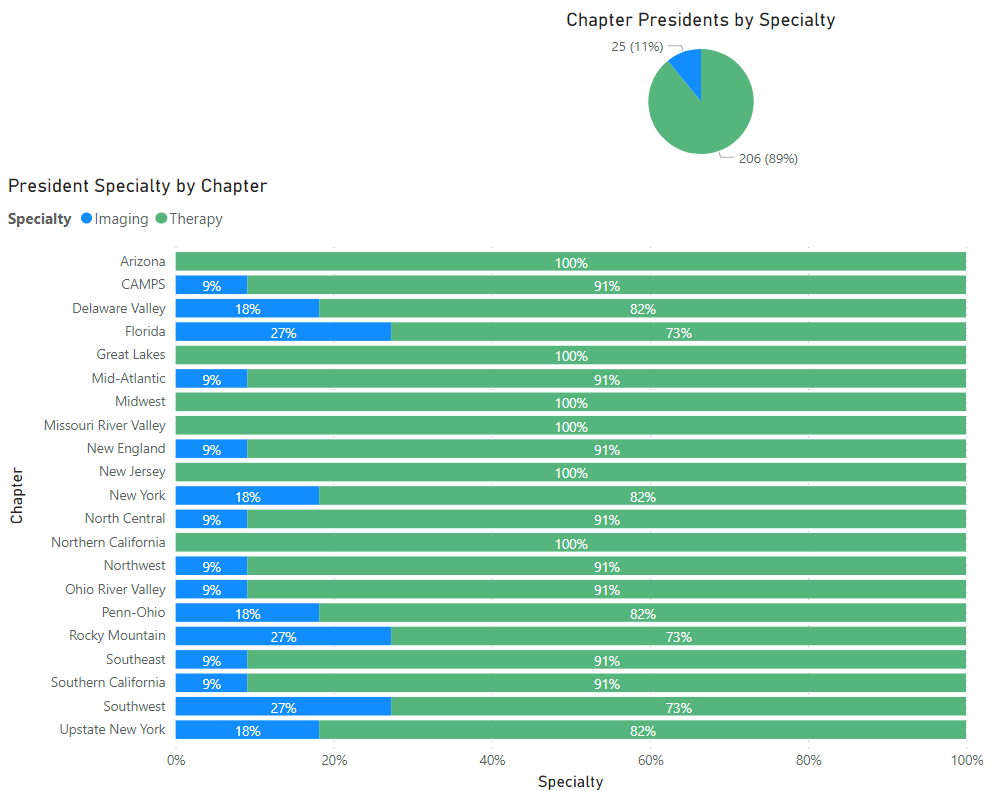


Figure 11: Primary specialty of chapter presidents by AAPM chapter from 2013-2023


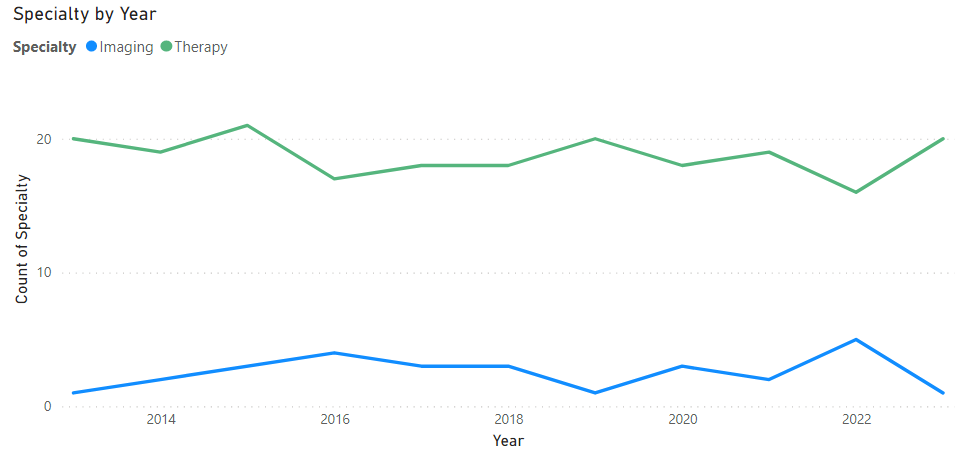


Figure 12: Chapter trends in primary specialty over time for chapter presidents

## Data by Board Representatives


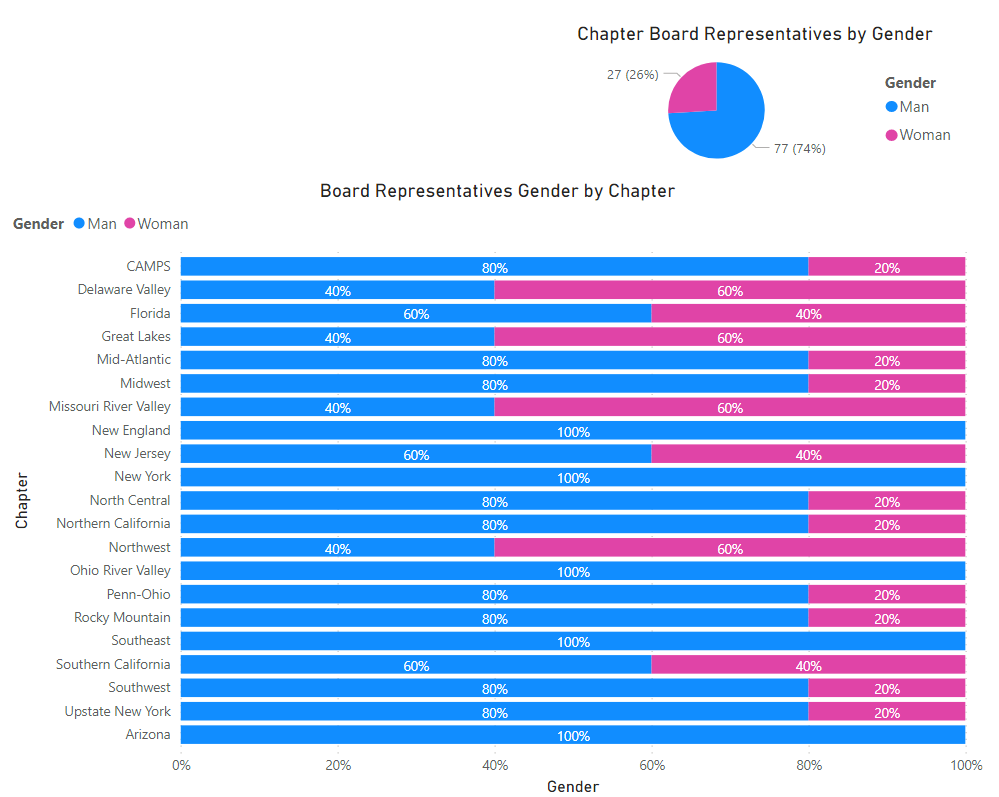


Figure 13: Gender of chapter board representatives by AAPM chapter from 2013-2023


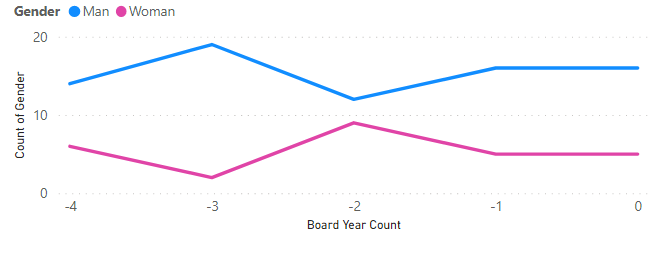


Figure 14: Chapter trends in gender over time for chapter board representatives. Since board members serve three-year terms with rotating starting dates depending on the chapter, the leaders were assigned numbers starting with 0 as the current representative and antecedent representatives are listed based on past term to display trends chronologically.


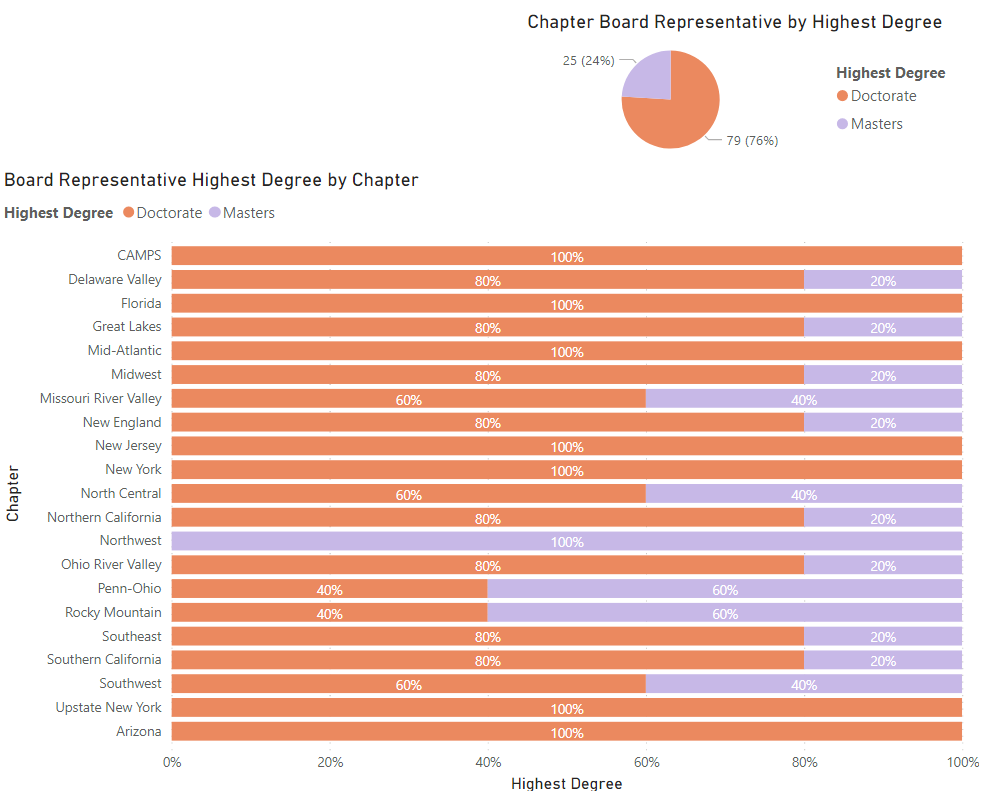


Figure 15: Highest degree of chapter board representatives by AAPM chapter from 2013-2023


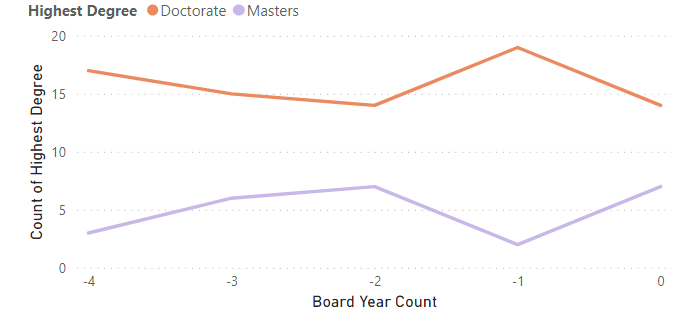


Figure 16: Chapter trends in highest degree over time for chapter board representatives. Since board members serve three-year terms with rotating starting dates depending on the chapter, the leaders were assigned numbers starting with 0 as the current representative and antecedent representatives are listed based on past term to display trends chronologically.


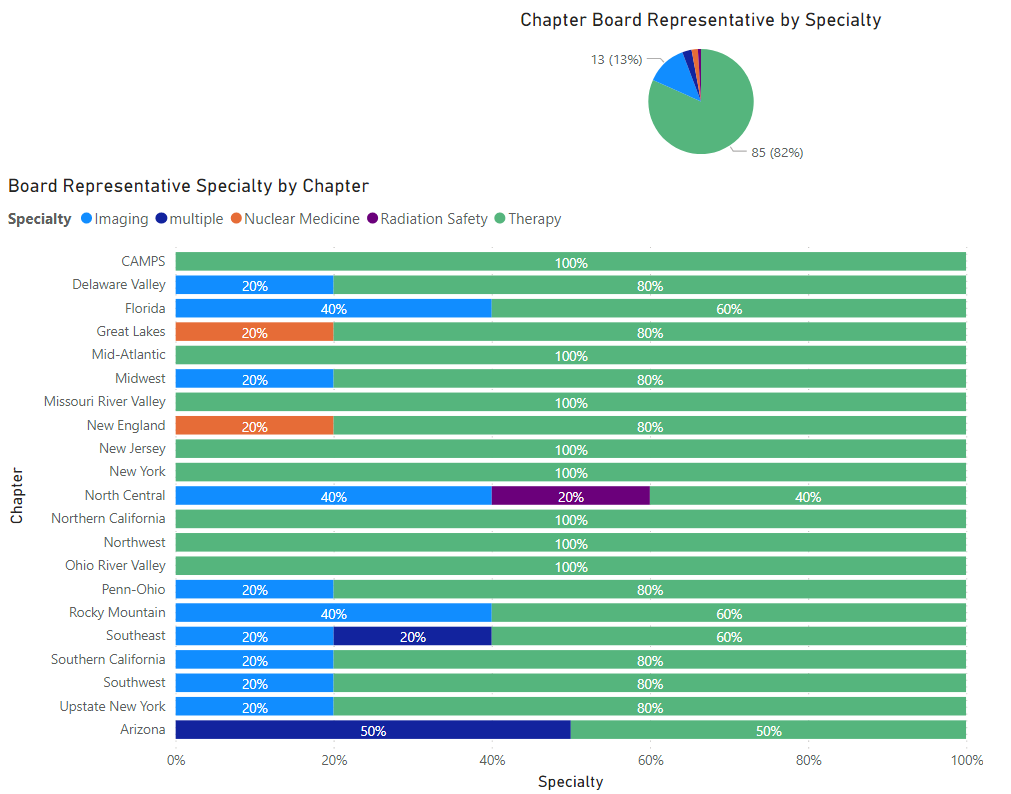


Figure 17: Primary specialty of chapter board representatives by AAPM chapter from 2013-2023


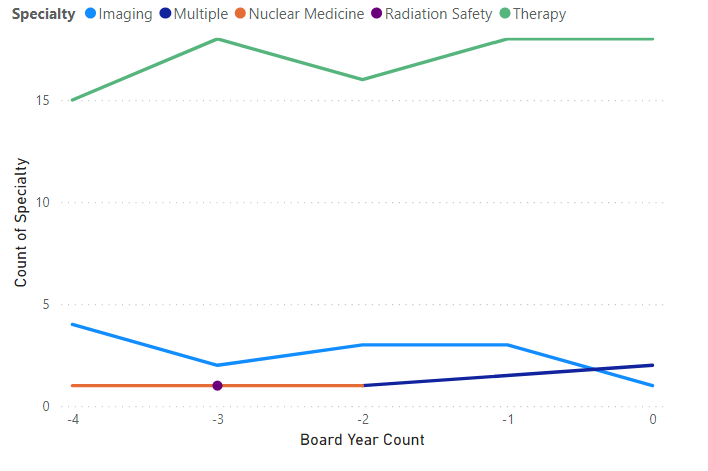


Figure 18: Chapter trends in primary specialty over time for chapter board representatives. Since board members serve three-year terms with rotating starting dates depending on the chapter, the leaders were assigned numbers starting with 0 as the current representative and antecedent representatives are listed based on past term to display trends chronologically.
